# Power to Punish Norm Violations Affects the Neural Processes of Fairness-Related Decision Making

**DOI:** 10.3389/fnbeh.2015.00344

**Published:** 2015-12-15

**Authors:** Xuemei Cheng, Li Zheng, Lin Li, Xiuyan Guo, Qianfeng Wang, Anton Lord, Zengxi Hu, Guang Yang

**Affiliations:** ^1^Shanghai Key Laboratory of Magnetic Resonance, Department of Physics, East China Normal UniversityShanghai, China; ^2^Key Laboratory of Brain Functional Genomics, Ministry of Education, Shanghai Key Laboratory of Brain Functional Genomics, School of Psychology and Cognitive Science, East China Normal UniversityShanghai, China; ^3^School of Psychology and Cognitive Science, East China Normal UniversityShanghai, China; ^4^Shanghai Key Laboratory of Magnetic Resonance and Key Laboratory of Brain Functional Genomics, Ministry of Education, Shanghai Key Laboratory of Brain Functional Genomics, School of Psychology and Cognitive Science, East China Normal UniversityShanghai, China; ^5^Clinical Affective Neuroimaging Laboratory, Leibniz Institute for NeurobiologyMagdeburg, Germany; ^6^School of Finance and Statistics, East China Normal UniversityShanghai, China

**Keywords:** punishment, the ultimatum game, the impunity game, fairness, decision making

## Abstract

Punishing norm violations is considered an important motive during rejection of unfair offers in the ultimatum game (UG). The present study investigates the impact of the power to punish norm violations on people’s responses to unfairness and associated neural correlates. In the UG condition participants had the power to punish norm violations, while an alternate condition, the impunity game (IG), was presented where participants had no power to punish norm violations since rejection only reduced the responder’s income to zero. Results showed that unfair offers were rejected more often in UG compared to IG. At the neural level, anterior insula and dorsal anterior cingulate cortex were more active when participants received and rejected unfair offers in both UG and IG. Moreover, greater dorsolateral prefrontal cortex activity was observed when participants rejected than accepted unfair offers in UG but not in IG. Ventromedial prefrontal cortex activation was higher in UG than IG when unfair offers were accepted as well as when rejecting unfair offers in IG as opposed to UG. Taken together, our results demonstrate that the power to punish norm violations affects not only people’s behavioral responses to unfairness but also the neural correlates of the fairness-related social decision-making process.

## Introduction

Humans are motivated by fairness norm during the decision-making process. When treated unfairly, they are willing to incur cost to punish norm violations. This behavior appears counter-intuitive given standard economic models which assume that humans will always favor the highest personal reward. However, empirical studies from both behavioral and neuroimaging fields have observed this irrational behavior by employing the ultimatum game (UG) (Güth et al., 1982; Sanfey et al., 2003). In UG, one player (proposer) makes a proposal of how to distribute a certain amount of money between him/herself and the other player (responder). The responder chooses to either accept the proposal, in which case both players get the amount specified in the proposal, or to reject the offer where both players receive nothing. Rejection of any offer is counter-intuitive considering standard economic models since any amount, no matter how unfair is still a net gain for the responder. However, nearly 50% of the unfair offers below 20% of the total amount were rejected by responders (Güth et al., 1982; Camerer and Thaler, 1995).

Rejection in UG has been considered to reflect individuals’ willingness to punish norm violations, which can be seen by contrasting UG with another economic game, the impunity game (IG) (Bolton and Zwick, 1995). The only difference between IG and UG is that if the responder rejects the offer, the proposer still gets the money while the responder gets nothing. Thus, rejection of unfair offers in IG does not punish norm-violating proposers. Previous studies have observed decreased rejection rates of unfair proposals in IG compared to UG, indicating that people’s responses to unfairness were affected by the power to punish norm violations (Bolton and Zwick, 1995; Güth and Huck, 1997; Takagishi et al., 2009; Yamagishi et al., 2009). However, how the neural mechanisms underlying the fairness-related decision-making process are affected by the power to punish norm violations is still unknown.

Many neuroscientific studies have investigated the neural processes of the responder’s responses to unfairness employing UG and identified the engagement of anterior insula (AI), dorsal anterior cingulate cortex (dACC), dorsolateral prefrontal cortex (DLPFC), and ventromedial prefrontal cortex (VMPFC) in the fairness-related decision-making process (Sanfey et al., 2003; Koenigs and Tranel, 2007; Güroğlu et al., 2010, 2011; Guo et al., 2013, 2014). AI and/or dACC have been suggested to be associated with detecting and responding to norm violations (Montague and Lohrenz, 2007; King-Casas et al., 2008; Güroğlu et al., 2010, 2011; Strobel et al., 2011). The increased activities of AI and dACC observed during receiving and rejecting unfair offers in UG might be related to the perception of fairness norm violation (Civai et al., 2012; Chang and Sanfey, 2013; Corradi-Dell’Acqua et al., 2013; Xiang et al., 2013; Guo et al., 2014). Left DLPFC was found to be more active during rejection than acceptance of unfair offers in UG (Güroğlu et al., 2010; Guo et al., 2013). Knoch et al. (2006) observed decreased rejection rates in UG after disrupting right DLPFC. Taken together, these results provide evidence for the involvement of DLPFC in overriding the desire to maximize one’s personal interest. Recent studies have also suggested that DLPFC plays a role in integrating information and selecting context-appropriate responses (Buckholtz et al., 2008; Buckholtz and Marois, 2012). Buckholtz and Marois (2012) suggests this “integration and selection” function could account for the involvement of DLPFC in rejection in UG. Thus, DLPFC might be engaged in selecting a context-appropriate response to unfairness in UG, with overriding the desire to maximize one’s personal interest being one of the underlying processes. Evidence from one brain lesion study demonstrated that patients with VMPFC damage made more rejections than healthy controls and patients with brain damage outside VMPFC (Koenigs and Tranel, 2007). Many studies have implicated VMPFC in the valuation of goods and integration of costs and benefits (de Quervain et al., 2004; Chib et al., 2009; Hare et al., 2009; Basten et al., 2010). Thus, damage to VMPFC might impede the process of evaluating financial gains in UG, which finally led to increased rejection behaviors (Moretti et al., 2009).

Takagishi et al. (2009) investigated the neural correlates of people’s responses to unfairness in IG. Increased AI and dACC activities were observed during rejection of unfair offers, indicating that the rejection behavior in IG was also associated with greater norm violations.

People make dozens of decisions in accordance with diverse and changing situations every day. In this study, we investigated the neural correlates of participants’ responses to unfairness when their power to punish norm violations was altered. UG and IG served as the context where participants did or did not have the power to punish norm violations respectively. In UG, participants’ rejection could punish proposers by reducing their income to zero. In contrast, rejection in IG did not punish the proposer, only the responder. Although the proposer was made aware of the rejection since they were not financially punished, this represented symbolic punishment only (Ule et al., 2009). We interchanged these two contexts dynamically throughout the experiment, so that participants might be more sensitive to any change in their power to punish norm violations. By adopting both UG and IG in one single study, we were able to directly compare neural responses from both conditions. Consistent with previous research, lower rejection rates were expected in IG compared with UG. At the neural level, increased activities of AI and/or dACC, which were associated with norm violations, were expected when participants received unfair offers and made rejections in both UG and IG (Sanfey et al., 2003; Takagishi et al., 2009). Regarding DLPFC, we made two predictions. First, stronger activation of DLPFC was expected during rejection relative to acceptance of unfair offers in both UG and IG based on its role in inhibiting the desire to maximize one’s personal interest (Knoch et al., 2006). Second, the dynamically switching contexts might make participants more sensitive to the change in their power to punish norm violations. Thus, in UG where participants had the power to punish norm-violating proposers, they might tend to select rejection as a default and context-appropriate response to unfair distributions (Buckholtz and Marois, 2012). Based on the “integration and selection” hypothesis which suggested DLPFC was associated with finding a context-appropriate response, we expected higher DLPFC activation during rejection than acceptance of unfair offers in UG. However, in IG rejection would only reduce participants’ own income to zero and inflict no punishments on proposers. It might not be easy to select an appropriate response since participants were neither willing to reject an offer which only penalized themselves nor accept an offer which they felt was unfair. Thus, DLPFC activity levels might be similar when accepting or rejecting unfair offers due to this trade-off process of selecting an appropriate response. VMPFC was expected to be more active when accepting unfair offers in UG than in IG. In contrast with IG, the rejection response which could inflict punishments on proposers might be treated as a default and context-appropriate reaction in UG, thus only offers which were considered to be desirable would be accepted. However, acceptance in IG seemed to be a forced choice due to participants’ inability to punish norm violations. Thus, the decision to accept an unfair proposal in UG might require a higher level of evaluating than the same decision in IG.

## Materials and Methods

### Participants

Thirty-seven right-handed volunteers took part in the study. Five participants were excluded from further statistical analyses. Of these five participants, one was excluded due a technical problem during scanning. Two had severe head motion (>3 mm or 3°). One did not accept any offers in IG and the last one missed more than 30% trials. The remaining 32 participants (23 females) had a mean age of 21.7 years (*SD* = 3.1 years) and normal or corrected-to normal vision. No abnormal neurological history was reported by any participant. Written informed consent was acquired from all participants before scanning. The study was approved by the Ethics Committee of the East China Normal University.

### Materials

Seventy-two face pictures (displayed as proposers), were selected from Chinese Facial Affective Picture System (Gong et al., 2011) and were randomly allocated to 2 (Context: UG vs. IG) ^∗^ 2 (Unfairness: Fair vs. Unfair) conditions. Half of the faces selected to represent the proposers were female. There were 36 pictures in each context with 12 pictures in fair trials and 24 in unfair trials. The emotion valence, arousal and attractiveness of pictures were counterbalanced across different conditions.

### Procedure

After giving informed consent, participants were told that they would take part in two economic games and the rule of each game. For the rule of UG, participants were told that they would receive a proposal about how to divide 50 RMB from a proposer. They would then respond to the offer and decide whether to accept or reject this proposal. If they accepted the proposal, both themselves and the proposer would get the money as suggested, however, if they rejected the proposal neither of them would get anything. The explanation of the rule of IG was the same with that of UG except the outcome of rejection. Participants were told that if they rejected the proposal, it had no influence on the proposer’s income but reduced their own to zero. However, the proposer would be informed of their decisions (either acceptance or rejection). Participants were also told that they would receive proposals from 72 different proposers whose proposals were obtained before the experiment. As for the payment, they were informed that several trials would be randomly selected after the games and that both themselves and the proposers would get paid according to their decisions. Finally, for each participant, 5% of their trials (four trials) were randomly selected. Participants were then shown these trials along with their responses and were paid accordingly. An additional 50 RMB was also given for their participation in the experiment.

**FIGURE 1:**
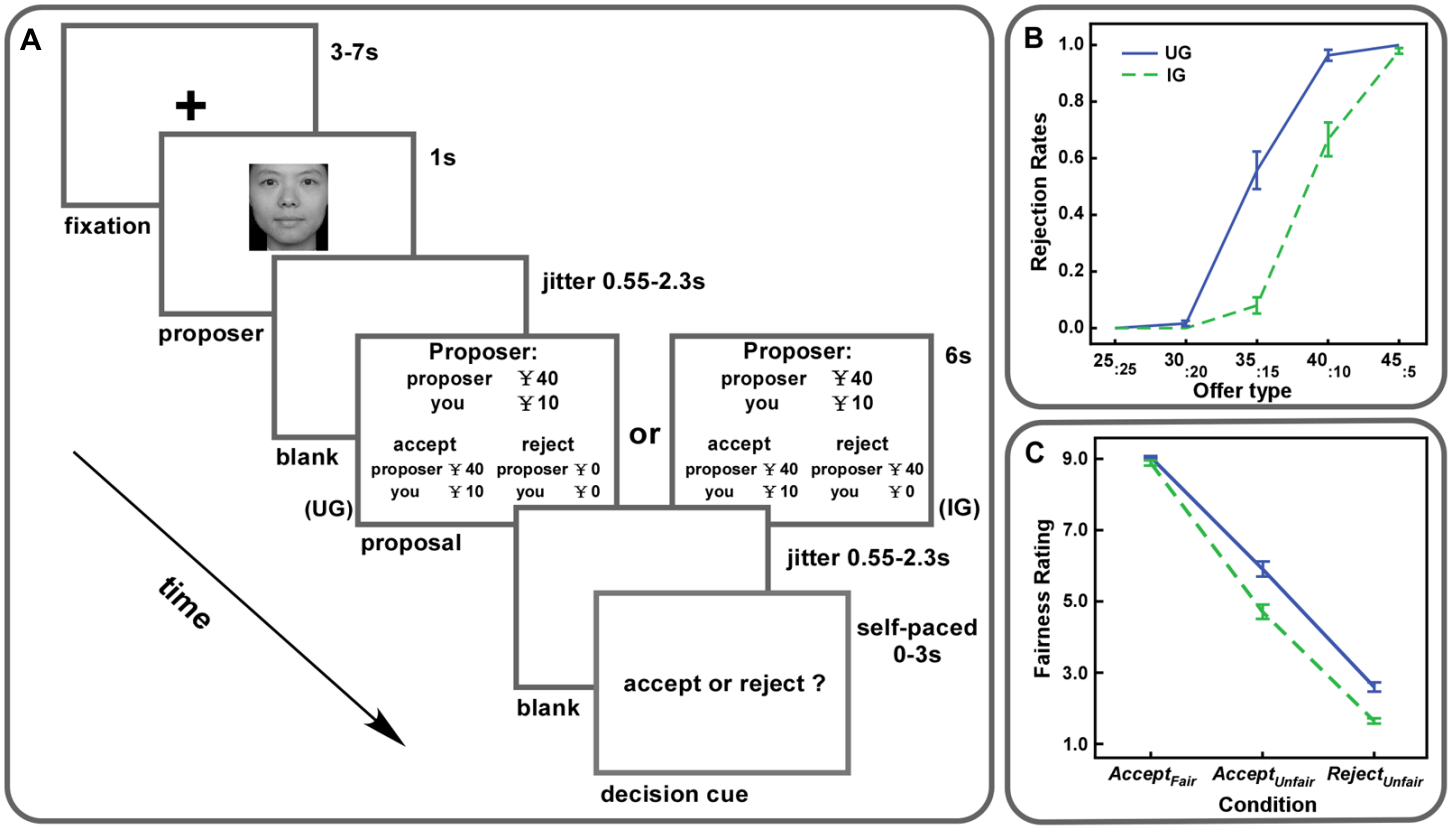
**(A)** Experiment procedure. Participants were scanned while they were playing the ultimatum game (UG) and the impunity game (IG) with each game containing 36 trials. Each trial involved splitting ¥50. In each game, fair offers (¥25:¥25) were given in 12 trials and unfair offers (six trials of ¥30:¥20, six trials of ¥35:¥15, six trials of ¥40:¥10, six trials of ¥45:¥5) were given in the remaining 24 trials. **(B)** Rejection rates of each offer type were plotted for both UG and IG. Error bars indicate SEM. **(C)** Fairness ratings for *Accept_Fair_*, *Accept_Unfair_*, and *Reject_Unfair_* trials were plotted for both UG and IG. Error bars indicate SEM.

Before scanning, participants practiced 24 trials of the task on a computer. After the practice, they completed 72 trials (**Figure 1A**) in the scanner. There were 36 trials in each context, including 12 fair trials (¥25:¥25) and 24 unfair trials. The unfair proposals could be ¥30:¥20, ¥35:¥15, ¥40:¥10, and ¥45:¥5, with each type having six trials. All the trials were presented in a random order. At the beginning of each trial, the proposer’s picture was presented for 1 s. After that, the proposal was shown for 6 s indicating the proposer’s offer and the present context. Then, a decision cue appeared on the screen and participants decided to either accept or reject this offer within 3 s. Participants were told to press the left button of the magnet-compatible button box with their right index fingers for acceptance, otherwise press the right button with their right middle fingers for rejection. As soon as they responded, the decision cue would disappear. The inter-stimulus intervals were jittered from 3 to 7 s. Two additional jittered blanks (550∼2300 ms) were set between the presentation of the proposer’s picture and the proposal and, between the proposal and the decision cue.

After scanning, the same stimuli including the offer and the contextual information (e.g., UG or IG) were presented again. Participants were asked to rate the fairness of the offer in each context on a 9-point Likert-type scale with 1 indicating extremely unfair and 9 indicating extremely fair. Participants’ generalized sense of power was assessed by the generalized version of sense of power scale (Anderson et al., 2012). There were eight items in the scale (e.g., ‘In my relationship with others, I can get people to listen to what I say’). Participants were asked to rate their agreement with each item on a 7-point scale from 1 (‘Strongly disagree’) to 7 (‘Strongly agree’).

### fMRI Image Acquisition and Analysis

Participants were scanned using a 3T Siemens scanner at the Shanghai Key Laboratory of Magnetic Resonance of East China Normal University. Anatomical images were acquired using a T1-weighted, multiplanar reconstruction (MPR) sequence (TR = 1900 ms, TE = 3.42 ms, 192 slices, slice thickness = 1 mm, FOV = 256 mm, matrix size = 256 ^∗^ 256) (Wang et al., 2015). After that, functional images with 35 slices were acquired using a gradient-echo echo-planar imaging (EPI) sequence (TR = 2200 ms, TE = 30 ms, FOV = 220 mm, matrix size = 64 ^∗^ 64, slice thickness = 3 mm, gap = 0.3 mm) (Wang et al., 2015).

Data preprocessing and statistical analyses were performed with the SPM8 software package (Wellcome Department of Cognitive Neurology, London). The first five functional images were discarded from each subject to allow scanner equilibrium effects. Then, all functional images were slice timing corrected, realigned, normalized into the MNI space (resampled at 2 mm ^∗^ 2 mm ^∗^ 2 mm voxels), and smoothed with an 8-mm full-width half maximum isotropic Gaussian kernel.

First-level analyses were then performed for each subject using an event-related design. We modeled the onset of the proposal for six types of events *Accept_Fair UG_* (accepted fair offers in UG, mean = 11.9 trials, maximum = 12 trials, minimum = 10 trials), *Accept_Unfair UG_* (accepted unfair offers in UG, mean = 8.7 trials, maximum = 14 trials, minimum = 6 trials), *Reject_Unfair UG_* (rejected unfair offers in UG, mean = 15.1 trials, maximum = 18 trials, minimum = 10 trials), *Accept_Fair IG_* (accepted fair offers in IG, mean = 11.8 trials, maximum = 12 trials, minimum = 10 trials), *Accept_Unfair IG_* (accepted unfair offers in IG, mean = 13.5 trials, maximum = 18 trials, minimum = 8 trials) and *Reject_Unfair IG_* (rejected unfair offers in IG, mean = 10.3 trials, maximum = 16 trials, minimum = 6 trials). Additional regressors of no interest were created for partner presentation, decision phase and trials with no responses. For partner presentation, we modeled the onset of the presentation of the proposer’s picture. For the decision phase, the onset of the decision cue was modeled for six types of events. For trials with no responses (i.e., trials where participants did not respond), we modeled the onset of the proposal and the onset of the decision cue. All the regressors were modeled with zero duration and convolved with a canonical hemodynamic response function (HRF). Additional regressors included in the design matrix comprised six realignment parameters and one overall mean during the whole phase. Low-frequency noise was filtered by applying a cutoff of 128 s in the models. Six contrast images (*Accept_Fair UG_*, *Accept_Unfair UG_*, *Reject_Unfair UG_*, *Accept_Fair IG_*, *Accept_Unfair IG_*_,_ and *Reject_Unfair IG_*) for proposal presentation were acquired for each subject at the first-level analysis. These images were then analyzed in a flexible factorial design at the second group level employing a random effects model.

A conjunction analysis using the conjunction null hypothesis (Nichols et al., 2005) was conducted first with the *(Unfair – Fair)_UG_* and *(Unfair – Fair)_IG_* contrasts to explore common brain regions activated by unfairness in both UG and IG. The *(Unfair – Fair)* contrast was calculated as (*Accept_Unfair_* + *Reject_Unfair_ – 2^∗^Accept_Fair_*). Then, the Unfairness (Unfair vs. Fair) ^∗^ Context (UG vs. IG) interaction defined by the *(Unfair – Fair)_UG_ – (Unfair – Fair)_IG_* and reverse contrasts were computed to assess the influence of the power to punish norm violations on unfairness perception. Next, a second conjunction analysis was conducted using the *(Reject_Unfair_ – Accept_Unfair_)_UG_* and *(Reject_Unfair_ – Accept_Unfair_)_IG_* contrasts to determine activated areas common to rejection of unfair offers in both UG and IG. The Response (Accept vs. Reject) ^∗^ Context (UG vs. IG) interaction defined by the *(Reject_Unfair_ – Accept_Unfair_)_UG_ – (Reject_Unfair_ – Accept_Unfair_)_IG_* and reverse contrasts were computed to identify brain areas showing modulation of the responder’s responses to unfair offers by the power to punish norm violations. Brain activations modulated by the power to punish norm violations were identified by the *(IG – UG)* and reverse contrasts. Additionally, correlation analyses between rejection rates and corresponding contrasts were performed to test for brain-behavior relations. We first correlated rejection rates of unfair offers in UG with the *(Unfair – Fair)_UG_* contrast. Then, a similar correlation was conducted between rejection rates in IG and the *(Unfair – Fair)_IG_* contrast. We also calculated the absolute difference in rejection rates between UG and IG for each subject and correlated them with the *(Unfair – Fair)_IG_ – (Unfair – Fair)_UG_* and *(Reject_Unfair_ – Accept_Unfair_)_IG_ – (Reject_Unfair_ – Accept_Unfair_)_UG_* contrasts respectively.

For all analyses, an initial voxel-level threshold of uncorrected *p* < 0.001 was used. Then for regions with *a priori* hypotheses, small volume correction (SVC) was applied for multiple comparisons. These regions include bilateral AI, dACC, DLPFC, and VMPFC. The MRIcro software^1^ was used to create masks required in the SVC procedure. The masks of insula and ACC were defined based on the automated anatomical labeling atlas (AAL) (Tzourio-Mazoyer et al., 2002). For DLPFC and VMPFC, the masks were made using a two stage process. First, we defined a sphere with the radius of 15mm and the center at the coordinates (left DLPFC, MNI -34 46 20; right DLPFC, MNI 39 37 26; VMPFC, MNI 2 41 -6) from previous studies (Sanfey et al., 2003; Baumgartner et al., 2011). Then these spheres were intersected with the corresponding Brodmann areas (DLPFC BA9, BA46; VMPFC BA10, BA11, BA24, BA25, BA32). Only activations surviving the voxel-level threshold of family-wise error (FWE) corrected *p* < 0.05 after SVC were reported in the results. For regions without *a priori* hypotheses, a cluster-level threshold of *p* < 0.05 after FWE correction for multiple comparisons across the whole brain was used. The MarsBaR toolbox^2^ was used to extract beta values when significant activations were observed.

## Results

### Behavioral Results

Rejection rates and fairness ratings were calculated with responded trials. Trials with no response of acceptance or rejection were removed from further analyses. We performed a 2 (Context: UG vs. IG) ^∗^ 5 (Offer type: ¥25:¥25 vs. ¥30:¥20 vs. ¥35:¥15 vs. ¥40:¥10 vs. ¥45:¥5) repeated measures ANOVA on rejection rates, which revealed both significant main effects of context, offer type and a significant interaction between them (*Fs* > 28.33, *ps* < 0.01). The results indicated that rejection rates were higher in UG than those in IG and increased with the level of unfairness. *Post hoc* paired *t*-tests demonstrated that offers of ¥35:¥15, ¥40:¥10, and ¥45:¥5 were rejected more frequently in UG than those in IG (*ts* > 2.10, *ps* < 0.05, **Figure 1B**).

For fairness ratings, we conducted a 2 (Context: UG vs. IG) ^∗^ 3 (Condition: *Accept_Fair_* vs. *Accept_Unfair_* vs. *Reject_Unfair_*) repeated measures ANOVA. Significant main effects of context and condition (*Fs* > 85.91, *ps* < 0.01) were observed. A significant interaction between context and condition was also found [*F*(2,62) = 37.55, *p* < 0.01]. Paired *t*-tests revealed that fairness ratings for *Accept_Fair_*_,_ *Accept_Unfair_* and *Reject_Unfair_* trials in IG were lower than those in UG (*ts* > 2.48, *ps* < 0.05). Moreover, the difference of fairness ratings for *Reject_Unfair_* trials between UG and IG was significantly smaller than that for *Accept_Unfair_* trials, but significantly larger than that for *Accept_Fair_* trials (*ts* > 2.32, *p* < 0.05, with sequential Bonferroni correction, **Figure 1C**).

### fMRI Results

#### The Impact of the Power to Punish Norm Violations on Unfairness Perception

The conjunction analysis using the *(Unfair – Fair)_UG_* and *(Unfair – Fair)_IG_* contrasts revealed that bilateral AI (SVC, MNI -28 22 0 and MNI 32 26 2, voxel-level FWE corrected *p* < 0.05), dACC (SVC, MNI -6 30 32 and MNI 10 20 26, voxel-level FWE corrected *p* < 0.05) and DLPFC (SVC, MNI -38 34 24 and MNI 42 36 26, voxel-level FWE corrected *p* < 0.05) were activated specifically when receiving unfair offers in both UG and IG. These results demonstrated that AI, dACC and DLPFC were more strongly activated when participants were treated unfairly regardless of if they had the power to punish norm violations or not. Additional activated brain regions were listed in Supplementary Table S1.

**FIGURE 2:**
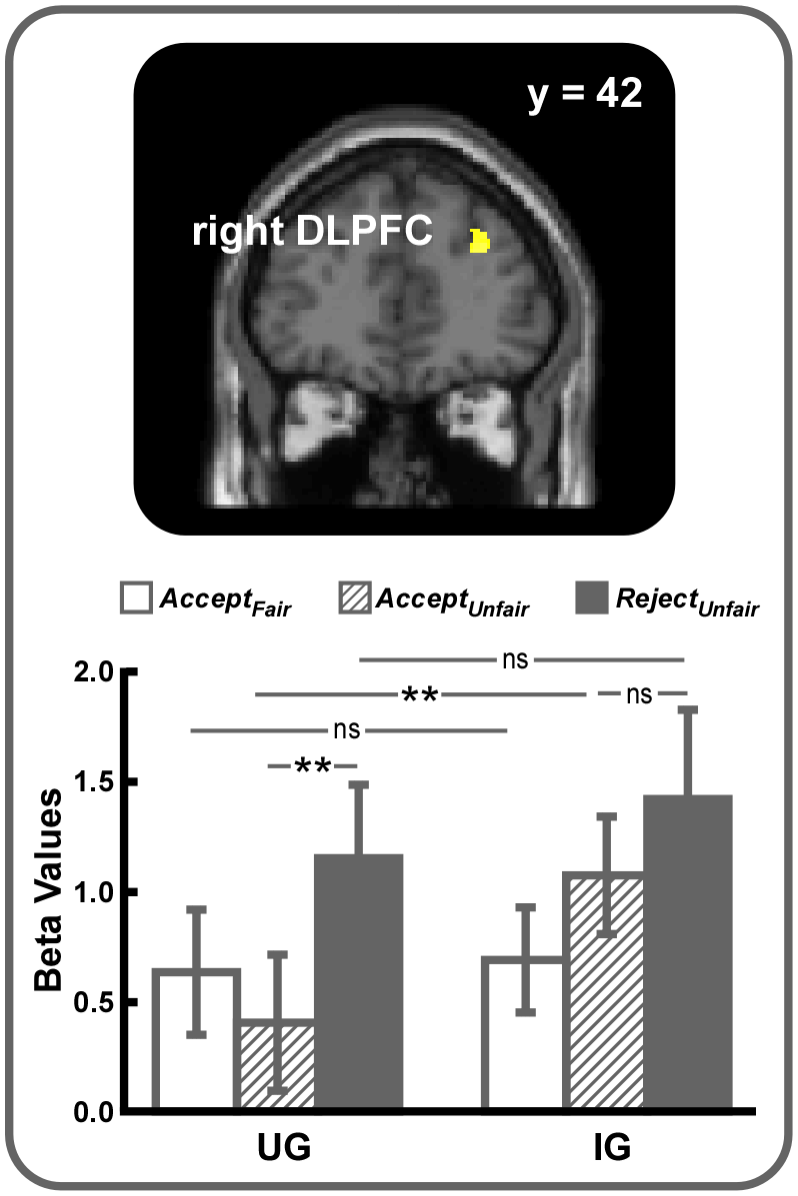
**Right DLPFC (small volume correction, MNI 30 42 28, voxel-level FWE corrected *p* < 0.05) was revealed in the *(Unfair – Fair)_IG_ – (Unfair– Fair)_UG_* contrast**. For display, a voxel-level threshold of uncorrected *p* < 0.001 was used. ^∗∗^*p* < 0.01, ns, not significant. Error bars indicated SEM.

Then, two contrasts were computed to explore how unfairness might interact with the power to punish norm violations. The *(Unfair – Fair)_UG_ – (Unfair – Fair)_IG_* contrast revealed no suprathreshold activations. However, right DLPFC activation (SVC, MNI 30 42 28, voxel-level FWE corrected *p* < 0.05) was observed in the reverse contrast (**Figure 2**). Further analyses on beta values showed that right DLPFC activity difference between UG and IG was significant in *Accept_Unfair_* condition (*t* = 2.99, *p* < 0.01) but not in *Accept_Fair_* (*t* = 0.20, *p* > 0.05) or *Reject_Unfair_* (*t* = 0.80, *p* > 0.05) conditions and the difference between acceptance and rejection of unfair offers was significant in UG (*t* = 2.99, *p* < 0.01) but not in IG (*t* = 1.02, *p* > 0.05), indicating that the difference of right DLPFC activity during acceptance of unfair offers between UG and IG might be mainly responsible for driving this interaction. Additional brain areas activated in the *(Unfair – Fair)_IG_ – (Unfair – Fair)_UG_* contrast were listed in Supplementary Table S2.

#### The Impact of the Power to Punish Norm Violations on Responses to Unfairness

**FIGURE 3:**
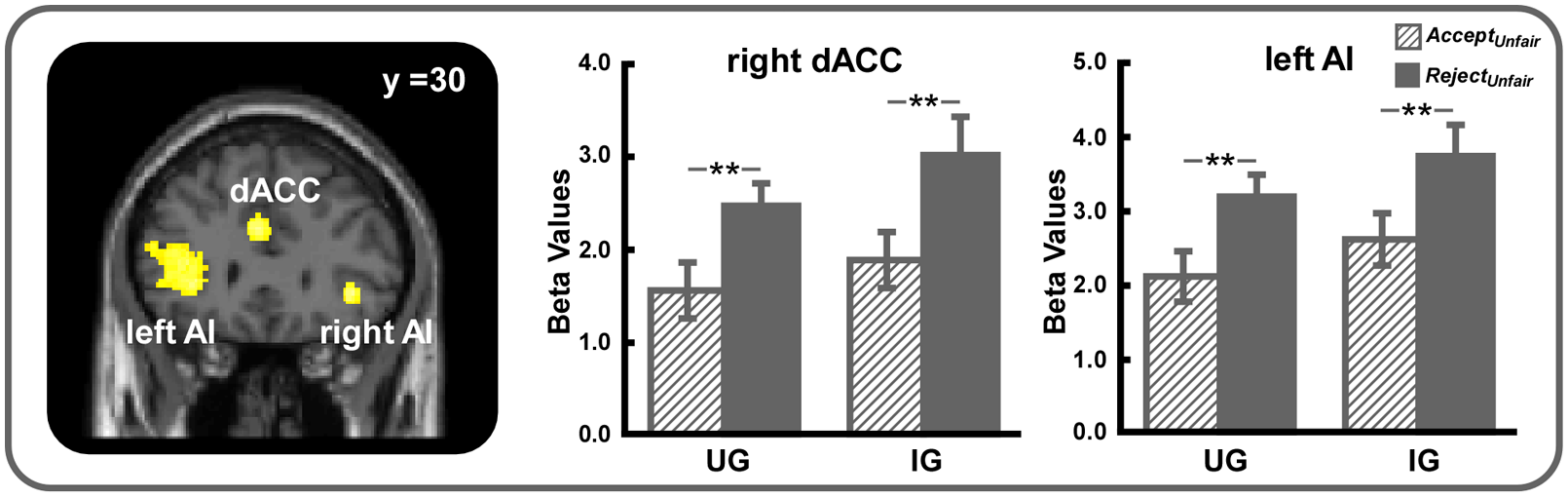
**Bilateral AI (small volume correction, MNI -32 22 4 and MNI 38 30 -2, voxel-level FWE corrected *p* < 0.05) and dACC (small volume correction, MNI -2 26 30 and MNI 8 20 26, voxel-level FWE corrected *p* < 0.05) were more strongly activated when unfair offers were rejected in both UG and IG**. For display, a voxel-level threshold of uncorrected *p* < 0.001 was used. ^∗∗^*p* < 0.01. Error bars indicated SEM.

The conjunction analysis using the *(Reject_Unfair_ – Accept_Unfair_)_UG_* and *(Reject_Unfair_ – Accept_Unfair_)_IG_* contrasts demonstrated that bilateral AI (SVC, MNI -32 22 4 and MNI 38 30 -2, voxel-level FWE corrected *p* < 0.05) and dACC (SVC, MNI -2 26 30 and MNI 8 20 26, voxel-level FWE corrected *p* < 0.05) were more active when unfair offers were rejected in both UG and IG (**Table 1**, **Figure 3**). Other regions activated under this contrast were listed in **Table 1**.

**Table 1:** Common areas activated by rejection of unfair offers in both ultimatum game (UG) and impunity game (IG).

		Peak Activation				
	Region	*X*	*Y*	*Z*	*t* Value	Voxels
**Conjunction analysis of *(Reject_Unfair_ – Accept_Unfair_)_UG_* and *(Reject_Unfair_ – Accept_Unfair_)_IG_***						
R	Cerebellum^∗∗^	28	-52	-28	5.01	1335
L	Inferior frontal gyrus^∗∗^	-40	38	10	4.75	2421
L	*AI^∗^*	-32	22	4	4.57	539
R	dACC^∗^	8	20	26	4.63	354
L		-2	26	30	4.48	
L	Thalamus^∗∗^	-16	-10	0	4.59	627
L	SupraMarginal gyrus^∗∗^	-60	-40	32	4.47	334
R	Middle occipital Gyrus^∗∗^	28	-80	14	4.43	599
L	Calcarine gyrus^∗∗^	2	-84	4	4.08	216
R	AI^∗^	38	30	-2	4.01	15

*L, left, R, right; coordinates (mm) were in MNI space*.

*A voxel-level threshold of uncorrected *p* < 0.001 was initially used. Then small volume correction was applied for *a priori* regions of interests and only activations surviving the voxel-level threshold of FWE corrected *p* < 0.05 were reported. For regions without *a priori* hypotheses, only activations surviving the cluster-level threshold of *p* < 0.05 after FWE correction for multiple comparisons across the whole brain were reported*.

*^∗^After small volume correction at voxel-level FWE corrected *p* < 0.05*.

*^∗∗^After whole brain correction at cluster-level FWE corrected *p* < 0.05*.

**FIGURE 4:**
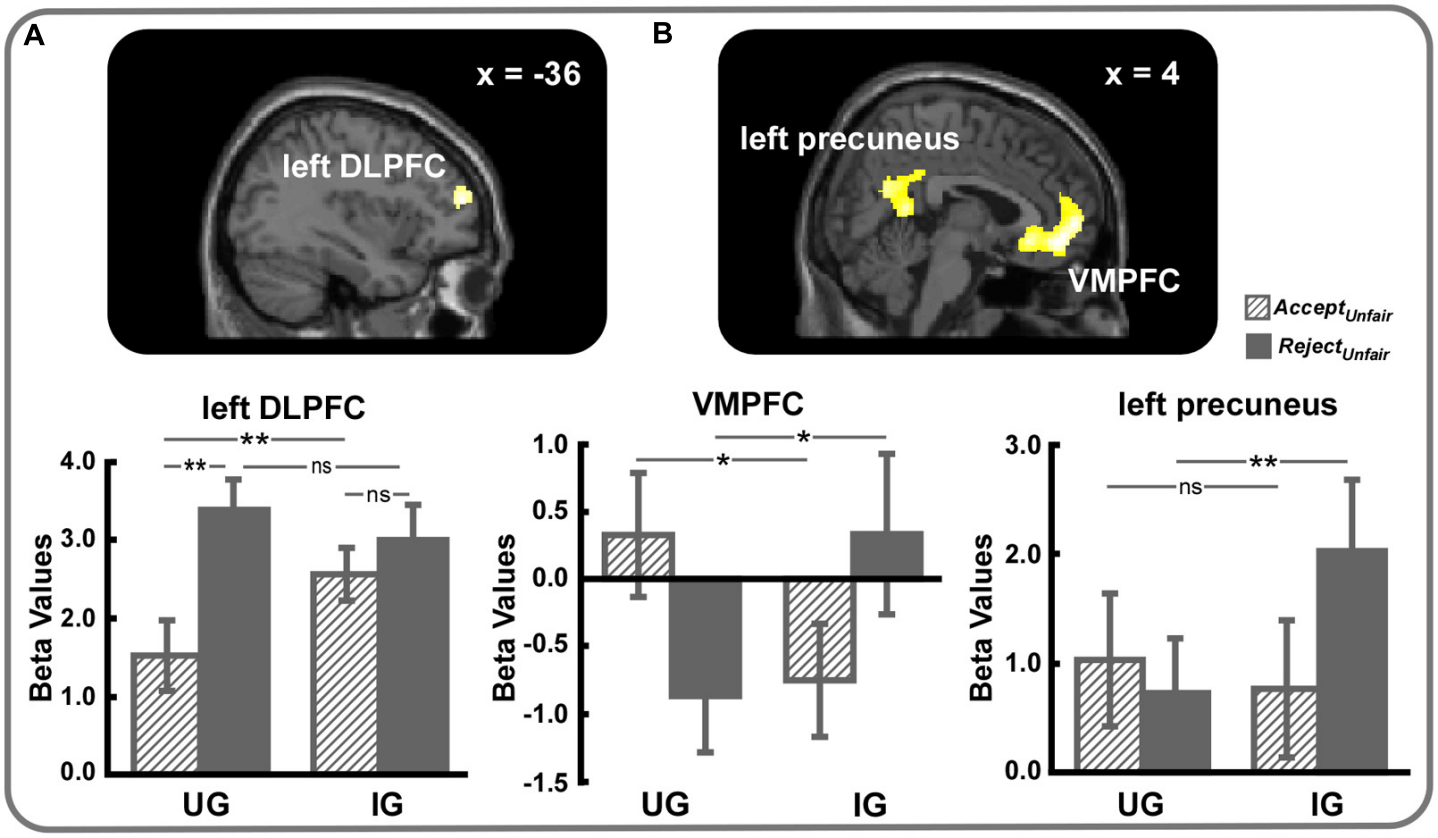
**(A)** Left DLPFC (small volume correction, MNI -36 52 16, voxel-level FWE corrected *p* < 0.05) and **(B)** VMPFC (small volume correction, MNI 4 50 -6, voxel-level FWE corrected *p* < 0.05) and left precuneus (MNI 0 -62 24, cluster-level FWE corrected *p* < 0.05) were engaged in the response ^∗^ context interaction. For display, a voxel-level threshold of uncorrected *p* < 0.001 was used. ^∗^*p* < 0.05, ^∗∗^*p* < 0.01, ns, not significant. Error bars indicated SEM.

Neural correlates of responses to unfairness modulated by the power to punish norm violations were identified by the response ^∗^ context interaction. The *(Reject_Unfair_ – Accept_Unfair_)_UG_ – (Reject_Unfair_ – Accept_Unfair_)_IG_* contrast revealed activation of left DLPFC (SVC, MNI -36 52 16, voxel-level FWE corrected *p* < 0.05) (**Table 2**, **Figure 4A**). Further analyses on beta values showed that left DLPFC was more active during rejection as opposed to acceptance of unfair offers in UG (*t* = 5.65, *p* < 0.01) but not in IG (*t* = 1.37, *p* > 0.05). Furthermore, acceptance of unfair offers activated more left DLPFC in IG than in UG (*t* = 3.04, *p* < 0.01), whereas rejection exhibited no left DLPFC activity difference between UG and IG (*t* = 1.03, *p* > 0.05).

**Table 2:** Regions showing Response (*Accept_Unfair_* vs. *Reject_Unfair_*) ^∗^ Context (UG vs. IG) interaction effect.

		Peak Activation				
	Region	*X*	*Y*	*Z*	*t* Value	Voxels
***(Reject_Unfair_ – Accept_Unfair_)_UG_ – (Reject_Unfair_ – Accept_Unfair_)_IG_***	
L	DLPFC^∗^	-36	52	16	3.99	124
***(Reject_Unfair_ – Accept_Unfair_)_IG_ – (Reject_Unfair_ – Accept_Unfair_)_UG_***	
L	Precuneus^∗∗^	0	-62	24	4.82	674
R	VMPFC^∗^	4	50	-6	4.81	927

*L, left, R, right; coordinates (mm) were in MNI space*.

*A voxel-level threshold of uncorrected *p* < 0.001 was initially used. Then small volume correction was applied for *a priori* regions of interests and only activations surviving the voxel-level threshold of FWE corrected *p* < 0.05 were reported. For regions without *a priori* hypotheses, only activations surviving the cluster-level threshold of *p* < 0.05 after FWE correction for multiple comparisons across the whole brain were reported*.

*^∗^After small volume correction at voxel-level FWE corrected *p* < 0.05*.

*^∗∗^After whole brain correction at cluster-level FWE corrected *p* < 0.05*.

Considering the reverse contrast, *(Reject_Unfair_ – Accept_Unfair_)_IG_ – (Reject_Unfair_ – Accept_Unfair_)_UG_*, we observed significant activations of left precuneus (MNI 0 -62 24, cluster-level FWE corrected *p* < 0.05) and VMPFC (SVC, MNI 4 50 -6, voxel-level FWE corrected *p* < 0.05) (**Table 2**, **Figure 4B**). As can be seen in **Figure 4**, VMPFC was more strongly activated during acceptance of unfair offers in UG than in IG (*t* = 2.56, *p* < 0.05) and rejection of unfair offers in IG than in UG (*t* = 2.08, *p* < 0.05). As for left precuneus, greater activity was detected when unfair offers were rejected in IG than in UG (*t* = 2.79, *p* < 0.01), whereas no significant difference was observed when unfair offers were accepted (*t* = 0.70, *p* > 0.05).

### Brain Activities Modulated by the Power to Punish Norm Violations

Stronger activations of bilateral AI (SVC, MNI -30 18 -2 and 36 24 -2, voxel-level FWE corrected *p* < 0.05), right dACC (SVC, MNI 8 28 28, voxel-level FWE corrected *p* < 0.05) and right DLPFC (SVC, MNI 26 34 32, voxel-level FWE corrected *p* < 0.05) were detected in IG than in UG. Additional activated brain areas were listed in Supplementary Table S3. The reverse contrast revealed no suprathreshold activation.

### Correlation Analyses

The correlation analysis between rejection rates and the *(Unfair – Fair)_UG_* contrast revealed that clusters located in bilateral AI (MNI -28 18 -4, *r* = 0.68, *p* < 0.01 and MNI 40 12 -10, *r* = 0.65, *p* < 0.01), bilateral dACC (MNI -6 4 30, *r* = 0.63, *p* < 0.01 and MNI 4 48 30, *r* = 0.61, *p* < 0.01) and left DLPFC (MNI -26 56 14, *r* = 0.61, *p* < 0.01) positively correlated with rejection rates in UG. All of these regions survived SVC at voxel-level FWE corrected *p* < 0.05. No significant correlation was observed between rejection rates in IG and the *(Unfair – Fair)_IG_* contrast. We also found no significant correlations between the *(Unfair – Fair)_IG_ – (Unfair – Fair)_UG_* contrast and the absolute difference in rejection rates between UG and IG. However, activation of VMPFC (SVC, MNI 12 42 -12, voxel-level FWE corrected *p* < 0.05) in the *(Reject_Unfair_ – Accept_Unfair_)_IG_ – (Reject_Unfair_ – Accept_Unfair_)_UG_* contrast was found to positively correlate with the absolute difference in rejection rates (*r* = 0.59, *p* < 0.01, Supplementary Figure S1A), indicating that participants with larger behavioral change recruited more VMPFC in the interaction. Specifically, we found that the absolute difference in rejection rates positively correlated with the beta value difference for VMPFC between the *Reject_UnfairIG_* and *Reject_UnfairUG_* condition (*r* = 0.52, *p* < 0.01, Supplementary Figure S1B), and marginally with the beta value difference for VMPFC between the *Accept_UnfairUG_* and *Accept_UnfairIG_* condition (*r* = 0.35, *p* = 0.052, Supplementary Figure S1C). No negative brain-behavior correlations were detected.

## Discussion

In the present study, we used UG and IG to investigate how people’s responses to unfairness could be affected by the power to punish norm violations and the underlying neural correlates. Behavioral results revealed that rejection rates in IG decreased to a large extent compared to UG, indicating that the power to punish norm violations indeed influenced participants’ responses to unfairness. In contrast with previous work (Sanfey et al., 2003; Yamagishi et al., 2009) unfair offers were rejected more often in our study, especially when the worst offers were proposed (1:9). This might be due to the switching contexts in our study which might make participants more sensitive to the change in their power to punish norm violations. Thus when participants could punish norm-violating proposers, they might take better advantage of their power, resulting in higher rejection rates in UG. As for IG, rejection rates were different across previous studies. Bolton and Zwick (1995) observed no rejections in IG, whereas Takagishi et al. (2009) and Yamagishi et al. (2009) observed approximately 40 and 50% of the most unfair offers were rejected respectively. Yamagishi et al. (2009) attributed this divergence to different instructions of these studies. In the study of Bolton and Zwick (1995), participants were only instructed to choose between two payoff options. However, in other studies, participants were explicitly told to either accept or reject the offer (Güth and Huck, 1997; Takagishi et al., 2009; Yamagishi et al., 2009; Ma et al., 2012). Phrasing instructions with words such as “accept” and “reject” might promote participants to consider the fairness of the offer, thus rejection in IG might reflect responders’ unwillingness to be insulted by unfair treatments (Yamagishi et al., 2009). Moreover, Ma et al. (2012) showed that ones’ own reputation was a motivating factor when rejecting unfair offers in IG, indicating that rejection could be a way to protect personal reputation or defend self-image (Güth and Huck, 1997; Ma et al., 2012). In our study, participants experienced two contexts simultaneously where they did or did not have the power to punish norm violations. Considering the concerns of protecting personal reputation and defending self-image, participants might behave in a way which was consistent with their reputation and coherently across contexts (Baumeister and Jones, 1978; Greenberg, 1990; Ferris et al., 2003; Wang and Cui, 2006). Thus, they might still reject unfair offers when they could only symbolically punish norm-violating proposers, similar to when they could punish the proposers. This might be the reason why the rejection rates for the most unfair offers in IG in our study were similar with those in UG but comparatively higher compared with other studies in which participants only experienced the IG context (Takagishi et al., 2009; Yamagishi et al., 2009).

At the neural level, in line with the studies of Sanfey et al. (2003) and Takagishi et al. (2009), increased activity within AI and dACC were observed when participants received and rejected unfair offers in both UG and IG. Expanding on these two studies, in our study it is possible to directly compare neural correlates underling people’s responses to unfairness in UG and IG, allowing us to assess the modulatory effects of the power to punish norm violations. DLPFC and VMPFC were found to be differently involved in the decision-making process in UG and IG. Specifically, DLPFC exhibited stronger activation during rejection than acceptance of unfair offers in UG but similarly strong activations in IG. VMPFC showed increased activity during acceptance of unfair offers in UG than in IG and during rejection of unfair offers in IG than in UG. Together, our results indicate that the neural processes of fairness-related decision-making are modulated by the power to punish norm violations.

Social norms play an important role in fostering social peace, stabilizing cooperation and enhancing prosperity (Buckholtz and Marois, 2012). Fairness is also a form of social norm. When fairness norm is violated, people are willing to punish norm violations at their own cost regardless of if their personal economic payoff is affected by norm violations or not (e.g., third-party punishment in which an uninvolved outside party incur cost to punish norm violations even though their economic payoff is not affected by the norm violations) (Fehr and Fischbacher, 2004; Henrich et al., 2006). In this study, AI and dACC were more active when participants received unfair offers in both UG and IG, which confirmed the role that AI and dACC played in detecting fairness norm violations (Civai et al., 2012; Chang and Sanfey, 2013; Xiang et al., 2013; Guo et al., 2014; Zheng et al., 2015). This result suggests that people perceive fairness norm violation irrespective of the power to punish norm violations. Moreover, greater AI and dACC activities were also observed during rejection of unfair offers, indicating that the rejection behavior was caused by greater norm violations (Sanfey et al., 2003; Civai et al., 2012; Corradi-Dell’Acqua et al., 2013; Guo et al., 2013). Interestingly, increased AI and dACC activities were also observed in IG compared to UG, indicating that the level of perceived norm violation might be enhanced when the power to punish norm violations was deprived.

Enhanced recruitment of left DLPFC was found during rejection than acceptance of unfair offers in UG, which was consistent with previous studies employing the UG paradigm (Güroğlu et al., 2010; Guo et al., 2013). Meanwhile, diminished left DLPFC activity difference between rejection and acceptance of unfair offers was observed in IG. Intriguingly, this pattern of activity was also exhibited by right DLPFC which was involved in the unfairness and context interaction. Based on the role of DLPFC in overriding the desire to maximize one’s personal interest (Knoch et al., 2006) and the “integration and selection” function (Buckholtz and Marois, 2012), we made two predictions about DLPFC activity. The main difference between these two predictions was the activity pattern of DLPFC during responses to unfairness in IG. According to the role of DLPFC in inhibiting the desire to maximize one’s personal interest (Knoch et al., 2006), greater DLPFC activity was expected when rejecting as opposed to accepting unfair offers in IG. Nevertheless, from the perspective of the “integration and selection” hypothesis, DLPFC activity increases when attempting to find a context-appropriate response (Buckholtz and Marois, 2012). In UG where participants had the power to punish norm-violating proposers, rejection might be selected as a default and context-appropriate response to unfairness (Buckholtz and Marois, 2012). However, in IG it might not be easy to select a context-appropriate response since participants were neither willing to reject an offer which only penalized themselves nor willing to accept an offer which they felt was unfair. Thus, this trade-off process of selecting an appropriate response might involve similarly strong DLPFC activities during both acceptance and rejection of unfair offers in IG. Our results seem to be more in accordance with the second prediction, indicating that the integration and selection function of DLPFC plays an important role in fairness-related decision making.

Our research also identified that the engagement of VMPFC in responses to unfairness was modulated by the power to punish norm violations. VMPFC has been suggested to be involved in the evaluation process, including valuation of goods and integration of costs and benefits (de Quervain et al., 2004; Chib et al., 2009; Hare et al., 2009; Basten et al., 2010). When participants had the power to punish norm violations, they might treat the rejection (i.e., punishment) as a default and context-appropriate reaction toward unfair offers. An acceptance reaction would occur only when an offer was evaluated as desirable. However, in IG when people had no power to punish norm violations, to accept an offer seemed to be a first-order choice which demands little evaluation process. Our results showed increased activity of VMPFC during acceptance of unfair offers in UG compared to IG, which was consistent with the assumption. As discussed earlier, the rejection behavior in IG which represented symbolic punishment might be evaluated and considered as a way to express the responder’s unwillingness to be insulted by unfair treatments and willingness to protect personal reputation or defend their self-image (Güth and Huck, 1997; Yamagishi et al., 2009; Ma et al., 2012). Thus, more evaluation process might be involved in rejecting an unfair offer in IG relative to a default rejection in UG, which was supported by our data. Additionally, the absolute difference in rejection rates between UG and IG was found to positively correlate with the difference of VMPFC activity during rejection of unfair offers between IG and UG, as well as during acceptance of unfair offers between UG and IG. The absolute difference in rejection rates between UG and IG (i.e., behavioral change) reflected the degree to which a participant was influenced by the power to punish norm violations. Thus, this result indicated that participants who were more affected by the power to punish norm violations might engage more evaluating process during accepting unfair offers when they could punish norm-violating proposers as well as rejecting unfair offers when they could only make symbolic punishment.

## Conclusion

The present study revealed that the power to punish norm violations affected people’s behavioral responses to unfairness and the underlying neural correlates. People rejected unfair offers more often when they could punish the proposers. AI and dACC were more active when people received unfair offers and gave rejection responses no matter they had the power to punish norm violations or not. Increased DLPFC activity was observed during rejection than acceptance of unfair offers when rejection could inflict punishments, whereas this difference diminished when the punishment was absence. VMPFC showed increased activity during acceptance of unfair offers when participants had the power to punish norm violations and during rejection when they did not. In summary, the power to punish norm violations plays an important role in people’s fairness-related social decision-making process and the mechanisms underlying rejections of unfair offers with/without punishments might be different.

## Conflict of Interest Statement

The authors declare that the research was conducted in the absence of any commercial or financial relationships that could be construed as a potential conflict of interest.

## Footnotes

1 http://www.mccauslandcenter.sc.edu/mricro/mricro/index.html

2 http://marsbar.sourceforge.net
