# Supplementary material for: Power to Punish Norm Violations Affects the Neural Processes of Fairness-Related Decision Making
Source: Front Behav Neurosci. 2015 Dec 15;9:344. doi: 10.3389/fnbeh.2015.00344 (PMC4678221; doi:10.3389/fnbeh.2015.00344)
Supplement: Supplementary file 1 [file Data_Sheet_1.DOCX]

***Supplementary Material***

**Power to punish norm violations affects the neural processes of fairness-related decision making**

Xuemei Cheng, Li Zheng, Lin Li, Xiuyan Guo^*^, Qianfeng Wang, Anton Lord, Zengxi Hu, Guang Yang^*^

*** Correspondence:** Xiuyan Guo: [xyguo@psy.ecnu.edu.cn](mailto:xyguo@psy.ecnu.edu.cn), [wlkc_xyguo@126.com](mailto:wlkc_xyguo@126.com)

Guang Yang: [gyang@phy.ecnu.edu.cn](mailto:gyang@phy.ecnu.edu.cn)

**1 Supplementary Tables**

We conducted a conjunction analysis using the *(Unfair – Fair)_UG_* and the *(Unfair – Fair)_IG_* contrasts to investigate common areas activated by unfair relative to fair offers in both UG and IG. The commonly activated areas were listed in **Table S1**.

**Table S1** Common areas activated by unfairness in both UG and IG

|  |  | Peak Activation | | |  |  |
| --- | --- | --- | --- | --- | --- | --- |
|  | Region | X | Y | Z | *t* Value | Voxels |
| ***Conjunction Analysis of (Unfair – Fair)_UG_ and (Unfair – Fair)_IG_*** | | | | | | |
| L | Supplementary Motor Area** | -2 | 22 | 48 | 11.71 | 64674 |
| L | *AI** | -28 | 22 | 0 | 9.2 | 657 |
| R |  | 32 | 26 | 2 | 7.39 | 442 |
| L | *dACC** | -6 | 30 | 32 | 7.27 | 365 |
| R |  | 10 | 20 | 26 | 4.02 |  |
| R | *DLPFC** | 42 | 36 | 26 | 6.69 | 227 |
| L |  | -38 | 34 | 24 | 6.31 | 65 |

*Note*. L = left, R = right; coordinates (mm) were in MNI space.

A voxel-level threshold of uncorrected *p* < 0.001 was initially used. Then small volume correction was applied for a priori regions of interests and only activations surviving the voxel-level threshold of FWE corrected *p* < 0.05 were reported. For regions without a priori hypotheses, only activations surviving the cluster-level threshold of *p* < 0.05 after FWE correction for multiple comparisons across the whole brain were reported.

*After small volume correction at voxel-level FWE corrected *p* < 0.05.

**After whole brain correction at cluster-level FWE corrected *p* < 0.05.

The *(Unfair – Fair)_UG_ – (Unfair – Fair)_IG_* and the reverse contrasts were computed to explore how unfairness might interact with the power to punish norm violations. Brain areas activated in these two contrasts were listed in **Table S2**.

**Table S2** Regions showing Unfairness (Unfair vs. Fair) * Context (UG vs. IG) interaction effect

|  |  | Peak Activation | | |  |  |
| --- | --- | --- | --- | --- | --- | --- |
|  | Region | X | Y | Z | *t* Value | Voxels |
| ***(Unfair – Fair)_UG_ – (Unfair– Fair)_IG_*** | | | | | | |
|  | No Regions |  |  |  |  |  |
| ***(Unfair – Fair)_IG_ – (Unfair– Fair)_UG_*** | | | | | | |
| L | Middle Occipital Gyrus** | -36 | -86 | 6 | 4.68 | 579 |
| L | Supplementary Motor Area** | -2 | -12 | 56 | 4.22 | 247 |
| R | DLPFC* | 30 | 42 | 28 | 3.61 | 31 |

*Note*. L = left, R = right; coordinates (mm) were in MNI space.

A voxel-level threshold of uncorrected *p* < 0.001 was initially used. Then small volume correction was applied for a priori regions of interests and only activations surviving the voxel-level threshold of FWE corrected *p* < 0.05 were reported. For regions without a priori hypotheses, only activations surviving the cluster-level threshold of *p* < 0.05 after FWE correction for multiple comparisons across the whole brain were reported.

*After small volume correction at voxel-level FWE corrected *p* < 0.05.

**After whole brain correction at cluster-level FWE corrected *p* < 0.05.

Brain activations modulated by the power to punish norm violations were identified by the *(IG – UG)* and the reverse contrasts and listed in **Table S3**.

**Table S3** Brain activities modulated by the power to punish norm violations

|  |  | Peak Activation | | |  |  |
| --- | --- | --- | --- | --- | --- | --- |
|  | Region | X | Y | Z | *t* Value | Voxels |
| ***IG – UG*** | | | | | | |
| L | AI* | -30 | 18 | -2 | 6.38 | 288 |
| R |  | 36 | 24 | -2 | 6.24 | 542 |
| R | Precentral Gyrus** | 44 | 6 | 36 | 6.33 | 3038 |
| R | *dACC** | 8 | 28 | 28 | 4.53 | 224 |
| R | Linual Gyrus** | 12 | -66 | 4 | 6.26 | 1138 |
| L | Superior Parietal Lobule** | -26 | -60 | 52 | 5.91 | 551 |
| L | Superior Occipital Gyrus** | -18 | -66 | 26 | 4.96 | 771 |
| R | Angular Gyrus** | 32 | -56 | 52 | 4.72 | 275 |
| L | Precentral Gyrus** | -42 | 0 | 36 | 4.2 | 260 |
| R | DLPFC* | 26 | 34 | 32 | 3.54 | 8 |
| ***UG – IG*** | | | | | | |
|  | No Regions |  |  |  |  |  |

*Note*. L = left, R = right; coordinates (mm) were in MNI space.

A voxel-level threshold of uncorrected *p* < 0.001 was initially used. Then small volume correction was applied for a priori regions of interests and only activations surviving the voxel-level threshold of FWE corrected *p* < 0.05 were reported. For regions without a priori hypotheses, only activations surviving the cluster-level threshold of *p* < 0.05 after FWE correction for multiple comparisons across the whole brain were reported.

*After small volume correction at voxel-level FWE corrected *p* < 0.05.

**After whole brain correction at cluster-level FWE corrected *p* < 0.05.

**2 Supplementary Figures**


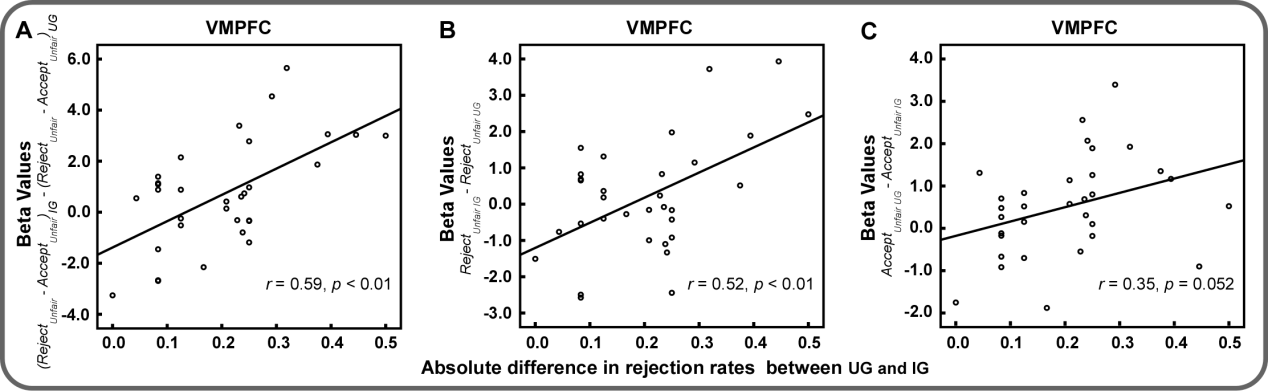


**Figure S1.** The absolute difference in rejection rates between UG and IG correlated with (**A**) activation of VMPFC (SVC, MNI 12 42 -12, voxel-level FWE corrected *p* < 0.05) in the *(Reject_Unfair_ – Accept_Unfair_)_IG_ – (Reject_Unfair_ – Accept_Unfair_)_UG_* contrast (**B**) the beta value difference for VMPFC between the *Reject_Unfair IG_* and *Reject_Unfair UG_* condition (**C**) the beta value difference for VMPFC between the *Accept_Unfair UG_* and *Accept_Unfair IG_* condition.
